# Improved Perinatal and Postpartum Human Immunodeficiency Virus Outcomes After Use of a Perinatal Care Coordination Team

**DOI:** 10.1093/ofid/ofz183

**Published:** 2019-04-09

**Authors:** Stephanie Hackett, Martina L Badell, Christina M Meade, Jennifer M Davis, Jeronia Blue, Lisa Curtin, Andres Camacho-Gonzalez, Ann Chahroudi, Rana Chakraborty, Minh Ly T Nguyen, Melody P Palmore, Anandi N Sheth

**Affiliations:** 1 Infectious Diseases Program, Grady Health System, Atlanta, Georgia; 2 Department of Gynecology and Obstetrics, Division of Maternal-Fetal Medicine, Atlanta, Georgia; 3 Department of Pediatrics, Division of Infectious Diseases, Atlanta, Georgia; 4 Department of Medicine, Division of Infectious Diseases, Atlanta, Georgia; 5 Emory University School of Medicine, Atlanta, Georgia

**Keywords:** contraception, HIV care continuum, maternal health, postpartum care, retention in care

## Abstract

In a high-volume clinic in the Southeastern United States, pregnant women living with human immunodeficiency virus (HIV) had improved HIV outcomes up to 6 months after delivery following the introduction of a multidisciplinary perinatal care coordination team.

## Teaser

Women living with HIV are at high risk for loss to follow-up after delivery. In this study, viral suppression at delivery, timely attendance of an HIV care visit after delivery, and viral suppression 6 months postpartum all significantly increased among women living with HIV after utilization of a multidisciplinary perinatal care coordination team in a large HIV clinic in Atlanta, Georgia.

Pregnancy presents a unique opportunity for women living with human immunodeficiency virus (HIV) (WLHIV) to adhere to antiretroviral therapy (ART) and achieve virologic suppression (VS) for their own health and to prevent perinatal HIV transmission. However, WLHIV are at dramatic risk for loss to follow-up and ART discontinuation after delivery [1–5]. Prompt transition to HIV care after delivery is associated with improved HIV outcomes up to 2 years post partum [3], but few other evidence-based practices exist to improve the HIV care continuum in postpartum WLHIV [6].

US HIV and obstetric guidelines emphasize the importance of care coordination between disciplines to optimize care [7, 8]. However, specific recommendations on how care coordination should be implemented are lacking. To address suboptimal HIV outcomes among pregnant and postpartum WLHIV in a high-volume, safety-net, hospital system in Atlanta, Georgia, we assembled a perinatal care coordination team in September 2015 and evaluated this program by comparing HIV outcomes before and after program implementation.

## METHODS

### Management of WLHIV Before the Perinatal Care Coordination Team Program

Before September 2015, pregnant WLHIV were referred from our outpatient HIV clinic, funded through the Ryan White HIV/AIDS Program, to the high-risk obstetric clinic located within the same healthcare system but in a separate clinical site. Women with newly diagnosed HIV during pregnancy were referred to the high-risk clinic from routine obstetric care. Clinical services in the high-risk obstetric clinic were provided by a maternal-fetal medicine specialist experienced in the care of pregnant WLHIV, an obstetric nurse, and a pediatric social worker; most women did not receive separate outpatient HIV clinic visits during pregnancy. Women were most often offered guideline-recommended protease inhibitor–based ART. Postpartum contraceptive counseling occurred as part of routine obstetric care, including counseling on the use of long-acting reversible contraceptive (LARC) methods; systems were not yet in place for routine immediate postpartum LARC placement. After delivery, women and infants were referred to enroll in the outpatient HIV clinic, which provided colocated pediatric services and used the same pediatric social worker. Case management services were also available in the adult HIV outpatient clinic.

### Program Description

In September 2015, a perinatal care coordination team was assembled, including obstetric (maternal-fetal medicine), adult, and pediatric HIV clinicians, an obstetric nurse, and a pediatric social worker; all members of the team were working in these roles during the preprogram period. The team (1) held monthly, in-person meetings to developed care plans for pregnant WLHIV from the time of prenatal care entry until transition to HIV care post partum, with efforts focused on women with viral nonsuppression, mental health and/or substance abuse disorders, or barriers to visit attendance, medication access, adherence, or medication tolerability; (2) strengthened direct communication and referrals between obstetric and HIV care providers; (3) provided an HIV clinician approximately once a month for direct consultation in the obstetric clinic; (4) streamlined HIV clinic enrollment and scheduling processes to allow for better coordination of postpartum visits for mother and baby; and (5) reviewed postpartum contraception plans to facilitate access and appropriate referrals for selected methods.

### Data Collection

We reviewed electronic medical records from entry into prenatal care until 24 months after delivery for WLHIV who delivered at ≥24 weeks gestation at Grady Memorial Hospital in Atlanta, between 1 January 2011 and 31 December 2017. Women who delivered at an outside hospital or intended to follow up for HIV care outside the healthcare system (9.7% of population) were excluded. Age, race/ethnicity, mode of HIV acquisition, year of HIV diagnosis, available ART history, CD4 cell count and HIV-1 RNA level (viral load [VL]), attendance at HIV care visits, gravidity, parity, mode of delivery, gestational age at delivery, number of prenatal care visits attended, birth outcome, attendance of postpartum obstetric follow-up visits, and contraception plan and use were recorded. For each HIV care visit after delivery, the VL, CD4 cell count, ART regimen, pregnancy status, contraception, and transfers of care were recorded. Research was approved by the Emory University Institutional Review Board and the Grady Research Oversight Committee.

### Outcomes

We compared the following outcomes before versus after program implementation (comparing women who delivered before vs after 1 September 2015): viral suppression (VS; HIV RNA <200 copies/mL) at delivery and 6 and 12 months after delivery; contraception provision at delivery; attendance at an HIV primary care visit within 90 days after delivery; and retention in HIV care 12 months after delivery (2 HIV care visits or VL measurements ≥90 days apart). The first HIV care visit or VL measurement after the postpartum obstetric visit was used to define a patient’s transition from obstetric to HIV care. If women did not have VL data, they were classified as having a VL ≥200 copies/mL.

### Analyses

We compared characteristics and outcomes in preprogram and postprogram groups using *t*, Wilcoxon rank sum, and χ^2^ tests, as appropriate. Multivariable logistic regression models were used to assess the association between program implementation and outcomes. Based on bivariate analysis and review of existing literature, models were adjusted for the following demographic and clinical variables: age, mode of HIV transmission, duration of HIV diagnosis, number of prenatal care visits, and ART use at pregnancy diagnosis. Postpartum outcomes models (attendance of an HIV care visit within 90 days after delivery, 6- and 12-month VS, and 12-month retention), also adjusted for VS at delivery and attendance of the postpartum obstetric visit. Analyses were conducted using SAS software (version 9.5); differences were considered statistically significant at *P* < .05.

## RESULTS

In all, 194 and 81 women delivered before and after the program, respectively. Women who delivered before the program were younger and had a more recent HIV diagnosis than those who delivered after the it (Table 1). Other demographic and clinical variables did not differ between groups.

**Table 1.** Patient Characteristics and Outcomes for Women Who Delivered Before or After Implementation of Perinatal Care Coordination Team Program

	WLHIV, No. (%)^a^ (N = 275)		
Characteristic	Before Implementation (n = 194)	After Implementation (n = 81)	*P* Value^b^
Age at delivery, mean (SD), y	27.4 (5.9)	29.1 (6.3)	.03^c^
Race/ethnicity			.15
African American (non-Hispanic)	160 (82)	60 (74)	
White (non-Hispanic)	12 (6)	4 (5)	
Hispanic	14 (7)	0 (0)	
Other/unknown	8 (4)	17 (21)	
No. of previous live births, mean (SD)	1.5 (1.5)	1.7 (1.5)	.29
Woman with congenital HIV infection	21 (11)	11 (14)	.52
HIV diagnosed during current pregnancy	38 (20)	14 (17)	.66
Time since HIV diagnosis, mean (SD), y	5.6 (6.3)	7.5 (7.7)	.03^c^
At pregnancy diagnosis			
Receiving ART	75 (40)	39 (48)	.21
CD4 cell count, mean (SD), cells/μL	417 (262)	427 (258)	.78
VS (HIV RNA <200 copies/mL)	72 (37)	36 (44)	.23
During pregnancy			
Gestational age at prenatal care entry, mean (SD), wk	17.1 (7.3)	16.2 (9.0)	.71
No. of prenatal care visits, mean (SD)	8.1 (3.7)	8.2 (3.6)	.94
VS for entire pregnancy	61 (32)	32 (40)	.20
VS for entire third trimester	121 (64)	56 (70)	.34
Delivery			
CD4 cell count, mean (SD), cells/μL	443 (239)	443 (200)	.99
VS	139 (72)	68 (84)	.03^c^
Cesarean delivery	96 (49)	35 (60)	.14
Gestational age >37 wk	165 (85)	65 (81)	.49
Any contraception plan at delivery	181 (94)	74 (93)	.70
Any contraception provision at delivery	121 (62)	55 (72)	.12
Either LARC or sterilization at delivery	46 (24)	35 (44)	<.001^c^
Post partum			
Attended obstetric visit	143 (73)	64 (83)	.08
Time to HIV care visit, d	147 (120)	74 (50)	<.001^c^
Attended HIV care visit within 90 d	57 (29)	50 (79)	<.001^c^
Any contraception provision within 90 d	138 (71)	62 (77)	.36^c^
Sterilization	28 (14)	11 (14)	.002
IUD or implant	25 (13)	24 (30)	
Injectable contraception	69 (36)	15 (19)	
Oral, patch, or ring	16 (8)	12 (15)	
None or condoms	56 (29)	19 (23)	
ART treatment interruption	104 (69)	31 (66)	.71
VS 6 mo after delivery	84 (43)	44 (59)	.02^c^
Retention in care 12 mo after delivery	88 (45)	27 (55)	.22
VS 12 mo after delivery	77 (40)	26 (53)	.10

Abbreviations: ART, antiretroviral therapy; HIV, human immunodeficiency virus; IUD, intrauterine device; LARC, long-acting reversible contraception; SD, standard deviation; VS, viral suppression; WLHIV, women living with HIV.

^a^Data represent no. (%) of WLHIV unless otherwise specified.

^b^Determined with χ^2^ or *t* tests, as appropriate.

^c^Significant at *P* < .05.

VS at delivery, HIV care visit attendance within 90 days after delivery, and VS at 6 months after delivery were all significantly improved in the postprogram group (all *P* < .05; Table 1). Maternal HIV care visits occurred on average 146 versus 73 days after delivery in the preprogram versus postprogram groups (*P* < .001). Although the proportions of women having any contraception plan and contraception provision at the time of delivery were high overall (94% and 64%, respectively) and did not differ between preprogram and postprogram groups, the proportion who elected to use either a LARC method or sterilization increased in the postprogram group (44% vs 24%; *P* < .001). Increases noted in 12-month retention and VS were not statistically significant. Three perinatal HIV infections occurred before versus none after program implementation.

In multivariable models, adjustment for age, mode of HIV transmission, time since HIV diagnosis, number of prenatal care visits, and ART use at pregnancy diagnosis, delivery after the program was associated with VS at delivery (odds ratio, 1.98; 95% confidence interval [CI], .91–4.29) and selection of LARC or sterilization for contraception (2.45; 1.37–4.41). In models that adjusted for the above factors as well as VS at delivery and attendance of the postpartum obstetric visit, delivery after the program was also significantly associated with HIV care visit attendance within 90 days after delivery (odds ratio, 9.46; 95% CI, 4.46–20.02) and VS 6 months after delivery (2.61; 1.31–5.22), but not retention in HIV care or VS 12 months after delivery (1.35 [95% CI, .68– 2.71] and 1.48 [.72–3.08], respectively).

## Discussion

We demonstrate improved HIV outcomes for WLHIV after the use of a multidisciplinary perinatal care coordination team. In a single-center study in South Carolina, provision of HIV care during pregnancy by maternal-fetal medicine specialists with HIV training was effective in reducing maternal VL at delivery, but outcomes beyond the initial postpartum period did not significantly improve [9]. In our clinical model, we previously observed that disengagement in HIV care was common after delivery and similar to reports from other US settings, despite providing HIV care during pregnancy that was similar to the South Carolina study model, even during the preprogram period [2–4, 10]. Our care coordination team therefore improves on previously reported models of HIV/obstetric care by extending improved outcomes to at least 6 months post partum [9].

Attendance at an HIV care visit within 90 days of delivery has been shown to predict retention in HIV care and VS post partum [2, 3]. Our monthly care plans aimed for timely HIV care transition by preemptively identifying operational barriers and aligning women’s postpartum HIV visits with their infants’ colocated pediatric visits, resulting in improved VS at 6 months, with trends toward improved 12-month outcomes. Our program highlights the importance of the multidisciplinary nature of the pregnancy care team to facilitate transition to and retention in HIV care after delivery.

In addition to improved HIV outcomes, we demonstrated increased provision and administration of LARC methods after implementation of the care coordination team, highlighting the potential for the team-approach to also address comprehensive reproductive health. LARCs have been designated as safe for WLHIV, with greater efficacy than other forms of reversible contraception [11–13] Despite the World Health Organization’s endorsement of LARCs for WLHIV since 2012 [13], studies have shown that uptake is lower among WLHIV than among women without HIV infection, and effective strategies to address this gap remain unknown [14, 15]. The care coordination team sought to improve overall efforts by the health system by facilitating continuity of family planning services in the postpartum period. In addition, owing in part to the gaps identified by the care coordination team, system-wide efforts were made to increase immediate postpartum access to LARC methods, which may have also played a role in improved uptake.

Programs to improve HIV outcomes often are associated with increased resources and cost [6, 9, 16]. Unique to our approach was that the care coordination team required commitment of minimal resources, to restructure individual team members’ time to provide care coordination during monthly meetings and periodic availability in the obstetric clinic. Meetings fostered improved communication between care team members and facilitated trust in joint medical decision making across team members with complementary expertise. Team management also facilitated prescribing of simpler and more tolerable ART regimens (including use of integrase inhibitors) and consideration of psychosocial factors in ART selection and management. These findings suggest that multidisciplinary care teams may reduce fragmentation of care between the antepartum and postpartum periods for patients with comorbid conditions and should be studied in diverse clinical settings with varying models of care.

Several lessons were learned from this program. First, simplification of enrollment procedures occurred clinic wide in 2016 as part of implementation of a rapid entry program [17]. Simplified clinic enrollment procedures complemented our program’s streamlined processes to improve postpartum linkage. Such initiatives should also be supported as a means to optimize perinatal outcomes. Second, the program commitment from leadership and individual team members was a key component of program success. Third, coordination meetings improved cross-disciplinary rapport among healthcare personnel, which fostered enhanced joint medical decision making with a focus on patient outcomes. Fourth, coordination of clinical visits for women and their infants was a key facilitator in improving visit attendance and therefore postpartum outcomes. A major challenge faced was clinic-wide turnover and provider shortages in the setting of higher-than-expected volumes of pregnant WLHIV, particularly at the end of the postimplementation period, which may have dampened the potential benefits of our program. This key challenge has been noted in implementation of other programs in our clinic [17] and across Ryan White–funded facilities [18] and must also be addressed for sustained improvements in perinatal outcomes for WLHIV.

There are limitations to this study. First, we relied on the accuracy and scope of electronic medical records. Thus, some socioeconomic variables could not be analyzed or compared in preprogram versus postprogram groups. Second, the results may be subject to temporal changes, which may have affected HIV outcomes. The relatively short time frame of this analysis helps to mitigate this limitation. Furthermore, we report a single-center program in a high-volume healthcare system. Care coordination teams should be further studied in other settings with varying models of care. Finally, the number of women who were able to achieve the 12-month outcomes was relatively small and may have precluded our ability to detect statistically significant changes at this time point. Nonetheless, our short-term findings are encouraging.

Despite challenges, use of a multidisciplinary perinatal care coordination team based in a high volume, Ryan White–funded clinic in the Southeastern United States, was feasible and associated with improved HIV outcomes up to 6 months after delivery. Timely transition to HIV care after delivery in the postprogram group highlights the potential benefits of perinatal care coordination teams to improve HIV outcomes 6 months or more after delivery.
